# Vaginal Lactoferrin Modulates PGE_2_, MMP-9, MMP-2, and TIMP-1 Amniotic Fluid Concentrations

**DOI:** 10.1155/2016/3648719

**Published:** 2016-10-31

**Authors:** Alessandro Trentini, Martina Maritati, Carlo Cervellati, Maria C. Manfrinato, Arianna Gonelli, Carlo A. Volta, Fortunato Vesce, Pantaleo Greco, Franco Dallocchio, Tiziana Bellini, Carlo Contini

**Affiliations:** ^1^Section of Medical Biochemistry, Molecular Biology and Genetics, Department of Biomedical and Specialist Surgical Sciences, University of Ferrara, 44121 Ferrara, Italy; ^2^Section of Dermatology and Infectious Diseases, Department of Medical Sciences, University of Ferrara, 44121 Ferrara, Italy; ^3^Department of Morphology, Surgery and Experimental Medicine, University of Ferrara, 44121 Ferrara, Italy; ^4^Section of Orthopedics, Obstetrics and Gynecology and Anesthesia and Resuscitation, Department of Morphology, Surgery and Experimental Medicine, University of Ferrara, 44121 Ferrara, Italy

## Abstract

Inflammation plays an important role in pregnancy, and cytokine and matrix metalloproteases (MMPs) imbalance has been associated with premature rupture of membranes and increased risk of preterm delivery. Previous studies have demonstrated that lactoferrin (LF), an iron-binding protein with anti-inflammatory properties, is able to decrease amniotic fluid (AF) levels of IL-6. Therefore, we aimed to evaluate the effect of vaginal LF administration on amniotic fluid PGE_2_ level and MMP-TIMP system in women undergoing genetic amniocentesis. One hundred and eleven women were randomly divided into controls (*n* = 57) or treated with LF 4 hours before amniocentesis (*n* = 54). Amniotic fluid PGE_2_, active MMP-9 and MMP-2, and TIMP-1 and TIMP-2 concentrations were determined by commercially available assays and the values were normalized by AF creatinine concentration. PGE_2_, active MMP-9, and its inhibitor TIMP-1 were lower in LF-treated group than in controls (*p* < 0.01, *p* < 0.005, and *p* < 0.001, resp.). Conversely, active MMP-2 (*p* < 0.0001) and MMP-2/TIMP-2 molar ratio (*p* < 0.001) were increased, whilst TIMP-2 was unchanged. Our data suggest that LF administration is able to modulate the inflammatory response following amniocentesis, which may counteract cytokine and prostanoid imbalance that leads to abortion. This trial is registered with Clinical Trial number NCT02695563.

## 1. Introduction

Inflammation plays an important role in pregnancy. Indeed, alternating proinflammatory and anti-inflammatory phases drive the implantation and the growth of the fetus, ending in the delivery of the baby at term by means of a sort of a final proinflammatory event [1]. However, an uncontrolled inflammatory response can lead to premature rupture of membranes (PROM), preterm PROM (PPROM), and preterm parturition [2]. Although the aforementioned complications might have a multifactorial etiology [3], a growing body of evidence suggests that intra-amniotic inflammation and infection, together with invasive diagnostic procedures, such as amniocentesis, may be important factors involved in the onset of pregnancy complications [2, 4, 5]. In this regard, it has recently been observed that a preexisting inflammatory state might predispose pregnant women undergoing amniocentesis to premature rupture of membranes within 48 hours from the procedure [6]. Furthermore, a higher AF concentration of IL-6 had already been reported in women with spontaneous abortion following amniocentesis [7]. In turn, IL-6 is able to stimulate the production of matrix metalloproteinases (MMPs) and prostaglandins (PGs) that act as effectors in the setting of PROM and preterm delivery [8].

In particular, MMP-9 and MMP-2, proteases belonging to the family of gelatinases, are able to degrade type IV collagen, highly expressed in the amniochorionic extracellular matrix [9], with a role in premature rupture of membranes. Indeed, a growing body of evidence suggests that MMP-9 is highly related to membrane rupture [9], with its overexpression at midtrimester being considered as a poor prognostic factor for term delivery [10]. In addition, a decrease or increase in AF concentration of TIMP-1, the preferential inhibitor of MMP-9, was reported to be associated with PROM [11, 12], collectively indicating an imbalance in the MMP-9/TIMP-1 ratio.

There are, however, conflicting results regarding MMP-2. Indeed, this enzyme has been found constitutively expressed in fetal membranes [13], although an increase at term labor has been reported [14]. Furthermore, MMP-2 has been implicated in PROM and PPROM with a supposed role in the damage and weakening of fetal membranes [15]. In fact, an increased MMP-2 proteolytic activity, not counterbalanced by its specific inhibitor TIMP-2, has been documented in patients in such conditions in relation to term labor, suggesting a disruption in the MMP/TIMP axis [11, 15]. On the other hand, several reports either found no relation with these pathological events [14, 16] or revealed a decrease in active MMP-2 with a concomitant increase in the levels of its inhibitor TIMP-2 associated with rupture of membranes [16, 17]. Considering that the fine regulation of MMPs activity occurs through the action of their specific endogenous inhibitors, the evaluation of the MMP/TIMP molar ratio may evidence an imbalance in the proteolytic activity of the enzymes which is not adequately counteracted by inhibition.

Besides MMPs, high AF concentrations of PGE_2_ have been found in patients with preterm labor either in the absence or in the presence of infection [18, 19].

Based on the above-mentioned evidence, the control of inflammation following amniocentesis might help to reduce the risk of the related complications. In a previous study [20], we observed that vaginal administration of lactoferrin (LF), a glycoprotein with bacteriostatic and anti-inflammatory properties [21, 22], 4 hours before amniocentesis led to a decrease in AF levels of IL-6.

Our aim, in the present study, was, therefore, to evaluate whether treatment with LF prior to amniocentesis might influence the production of active MMP-9, active MMP-2, their specific inhibitors, TIMP-1 and TIMP-2, and PGE_2_ in the AF of pregnant women undergoing midtrimester genetic amniocentesis.

## 2. Materials and Methods

### 2.1. Study Design and Amniotic Fluid Collection

One hundred and eleven pregnant women (mean age: 36.4 ± 4.4 years), undergoing genetic amniocentesis within the 16th–18th gestational weeks, were enrolled in a prospective clinical study carried out in the Obstetric Unit, University of Ferrara, from January 2014 to March 2015. The inclusion criteria were singleton gestation and maternal age as the only indication to fetal karyotyping. The exclusion criteria were consumption of drugs interfering with the immune system, previous miscarriages, and pregnancy at risk for maternal or fetal disease. Since lactose is one of the excipients in the drug tablets, subjects suffering from lactose intolerance were excluded.

Eligible patients were randomly assigned, in a 1 : 1 ratio, with a random number table to receive a vaginal compound containing 300 mg of lactoferrin (Difesan, Progine Farmaceutici, Firenze, Italy) 4 hours before amniocentesis (treated 4 hrs, *n* = 54) or were untreated (controls, *n* = 57). Obstetricians and research assistants were blinded to group assignment. Amniotic fluid samples were obtained by transabdominal amniocentesis and the sample not required for clinical purposes was centrifuged at 3000 rpm for 10 minutes at 4°C to remove particulate material. The supernatants were then aliquoted and stored at −80°C until assay. No abnormalities were revealed by genetic analysis.

This study conforms to The Code of Ethics of the World Medical Association (Declaration of Helsinki) and was conducted according to the guidelines for Good Clinical Practice (European Medicines Agency). The study was approved by The Local Ethics Committee and written informed consent was obtained from each patient prior to inclusion in the study. No identifying information was available to the authors of the study in order to protect the anonymity of the patients.

The full version of the methods used for the determination of active MMP-9, active MMP-2, and PGE_2_ has been included as supplemental material (see Supplemental Methods in Supplementary Material available online at http://dx.doi.org/10.1155/2016/3648719).

### 2.2. Assay of Active MMP-9

The levels of active MMP-9 in the AF were determined by a commercially available activity assay kit (Fluorokine E, human active MMP-9 fluorescent assay, R&D Systems, Minneapolis, USA). Following the manufacturer's instructions, samples were diluted 2 times with the calibrator diluent RD5-24, provided in the kit, and the concentration of active MMP-9 was determined by interpolation from a standard curve in the range 16–0.25 ng/mL. According to the manufacturer's instructions, the lower limit of detection was 0.005 ng/mL, the range of intra-assay CV was 3.9–4.8%, and the range of interassay CV was 8.0–9.3%.

### 2.3. Assay of Active MMP-2

Active MMP-2 concentration in the AF was determined by means of a commercially available activity assay kit (Matrix Metalloproteinase-2 Biotrak activity assay system, RPN2631, GE Healthcare, Milan, Italy). All reagents and standards were included in the kits and samples were assayed in accordance with the manufacturer's manual. Samples were diluted 2 times and the concentration of active MMP-2 was determined by interpolation with a standard curve in the range 4–0.125 ng/mL. The lower limit of quantification was assumed at 0.125 ng/mL, the range of intra-assay CV was 4.4–7.0%, and the range of interassay CV was 16.9–18.5%.

### 2.4. TIMP-1 and TIMP-2 Assay

Amniotic fluid concentrations of TIMP-1 and TIMP-2 were measured using commercially available “sandwich” ELISA kits (Tissue inhibitor of metalloproteinases-1, RPN2611 and Tissue inhibitor of metalloproteinases-2, RPN2618; GE Healthcare, Milan, Italy) according to the manufacturer's instructions. For the analysis of TIMP-1, amniotic fluids were diluted 40 times and the limit of detection was 1.25 ng/mL. For the analysis of TIMP-2, amniotic fluids were diluted 2 times and the limit of detection was 3 ng/mL.

### 2.5. PGE_2_ Assay

Amniotic fluid PGE_2_ was measured by means of a commercially available competitive enzyme immunoassay (Prostaglandin E_2_ EIA Kit-Monoclonal, Cat. no. 514010, Cayman Chemical Company, Ann Arbor, USA) following the manufacturer's instructions. Amniotic fluid samples were diluted 2 times and the concentration of PGE_2_ present in the sample was determined by interpolation with the standard curve in the range 1000–7.8 pg/mL using a four-parameter logistic fit (GraphPad Prism version 6.00 for Windows, GraphPad Software, La Jolla, California, USA). The detection limit was 15 pg/mL, the range of intra-assay CV was 3.7–10.1%, and the range of interassay CV was 6.4–20.9%.

### 2.6. Creatinine Assay

Creatinine levels were measured by using the alkaline picrate method. Briefly, 200 *μ*L of alkaline picrate solution, containing 30 mM of picric acid (Cat. no. 197378, Sigma-Aldrich, Milan, Italy) and 0.166 M NaOH, was added to 50 *μ*L of sample or creatinine standards in the range 0.1–0.0031 mg/mL and incubated in the dark for 30 minutes at room temperature with gentle shaking. The absorbance of the color developed by the reaction was measured at 492 nm in a Tecan Infinite M200 microplate reader (Tecan Group Ltd., Männedorf, Switzerland) and the amount of creatinine present in the sample was calculated by interpolation with the standard curve.

### 2.7. Statistical Analysis

The normality distribution of the variables was checked using the Kolmogorov-Smirnov test. Since the variables were not normally distributed, they were shown as median (interquartile range) and group comparisons were performed using the Mann-Whitney *U* test. Categorical variables were shown as numbers and percentages, and differences in frequency distributions were examined by means of the chi-square test. All analyses were performed using SPSS 21.0 for windows (SPSS Inc., Chicago, Illinois, USA). A *p* < 0.05 was considered statistically significant.

## 3. Results

Maternal and clinical characteristics of the study population (*n* = 111) are summarized in Table 1. No significant difference regarding maternal age, gestational age at amniocentesis, and ethnicity was found between the two groups. Interestingly, there were significantly higher values of creatinine in patients treated with lactoferrin compared to controls (Table 1, *p* < 0.0001). In order to avoid concentration artifacts, values of prostaglandin E_2_, active MMP-9, active MMP-2, TIMP-1, and TIMP-2 were normalized by the creatinine concentration and used for the statistical analysis.

**Figure 1:**
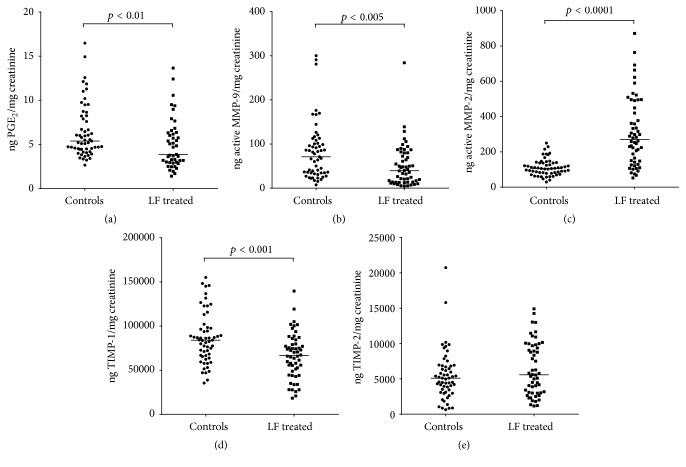
Concentrations of the inflammation markers measured in the amniotic fluids of controls and patients treated with lactoferrin 4 hours before amniocentesis. Values were normalized for the creatinine concentration and are presented as ng/mg creatinine. The Mann-Whitney *U* test was used for the comparison between the groups. Significant lower levels of PGE_2_ ((a), median [interquartile range] controls: 5.38 [4.44–8.24]; LF treated: 3.87 [2.97–6.33], *p* < 0.01), active MMP-9 ((b), controls: 71.02 [34.51–105.02]; LF treated: 42.67 [13.66–81.12], *p* < 0.005), and TIMP-1 ((d), controls: 84047 [66028–97949]; LF treated: 65952 [44201–82110], *p* < 0.001) were found in lactoferrin-treated patients compared to controls. Increased levels of active MMP-2 ((c), controls: 105.67 [80.61–137.17]; LF treated: 282.90 [127.40–492.71], *p* < 0.0001) were found in patients treated with lactoferrin whereas the levels of TIMP-2 were comparable ((e), controls: 5098 [3276–6829]; LF treated: 6266 [4031–9959], *p* = 0.233). In all the panels, the line between the data represents the median. PGE_2_: prostaglandin E_2_; MMP-9: matrix metalloproteinase-9; MMP-2: matrix metalloproteinase-2; TIMP-1: tissue inhibitor of metalloproteinase-1; TIMP-2: tissue inhibitor of metalloproteinase-2.

**Figure 2:**
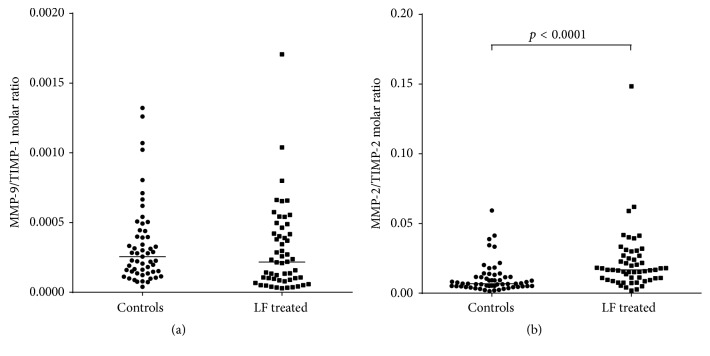
Evaluation of MMP/TIMP molar ratio in controls and patients treated with lactoferrin. MMP-9/TIMP-1 molar ratio was not different in controls and patients treated with lactoferrin 4 hours before amniocentesis (a), whereas we observed an increase in the MMP-2/TIMP-2 ratio after treatment with lactoferrin ((b), *p* < 0.0001). It is noteworthy that the ratio was below 1. In all the panels, the line between the data represents the median. MMP-9: matrix metalloproteinase-9; MMP-2: matrix metalloproteinase-2; TIMP-1: tissue inhibitor of metalloproteinase-1; TIMP-2: tissue inhibitor of metalloproteinase-2.

As reported in Figure 1, patients treated with lactoferrin 4 hours prior to amniocentesis showed a significant decrease in the levels of PGE_2_ ((a) controls: 5.3 (4.4–8.2) pg/mg creatinine; LF treated: 3.8 (2.9–6.3) pg/mg creatinine; *p* < 0.01), active MMP-9 ((b) controls: 71.0 (34.5–105.0) ng/mg creatinine; LF treated: 39.6 (13.7–79.9) ng/mg creatinine; *p* < 0.005), and TIMP-1 ((d) controls: 84048 (66029–97949) ng/mg creatinine; LF treated: 66579 (47716–80953) ng/mg creatinine; *p* < 0.001) compared to the controls. On the other hand, increased production of active MMP-2 was observed in the AF of patients treated with lactoferrin compared to controls ((c) controls: 105.7 (80.6–137.2) ng/mg creatinine; LF treated: 270.6 (146.6–459.5) ng/mg creatinine; *p* < 0.0001), whereas the TIMP-2 concentration did not differ between the two groups ((e) controls: 5098 (3277–6830) ng/mg creatinine; LF treated: 5568 (3010–9718) ng/mg creatinine; *p* = 0.235). Amniotic fluid MMP-9/TIMP-1 and MMP-2/TIMP-2 molar ratios were then calculated in order to identify possible imbalances in the proteolytic activity of the enzymes. As reported in Figure 2, the values were below 1 for both ratios, suggesting a control of the proteolysis. Although no difference was observed in the MMP-9/TIMP-1 molar ratio between controls and patients treated with lactoferrin (Figure 2(a): controls: 0.00025 (0.00014–0.00044); LF treated: 0.00022 (0.00008–0.00043); *p* = 0.183), a significant increase in MMP-2/TIMP-2 molar ratio was found following treatment with lactoferrin (Figure 2(b): controls: 0.0067 (0.0050–0.0112); LF treated: 0.0167 (0.0104–0.0268); *p* < 0.0001).

Neither maternal nor gestational age was seen to affect the variables (data not shown).

## 4. Discussion

Although a proinflammatory environment is necessary for both implantation and physiological birth, excessive inflammation pregnancy complications may lead to miscarriage or preterm birth [23]. Both microbial-associated and sterile intra-amniotic inflammation (IAI) are involved, with the sterile type assuming even greater importance [24]. In particular, sterile IAI can be a preexisting condition or can be either triggered or aggravated by invasive diagnostic procedures such as amniocentesis. Consistently, a recent study [6] suggested that preexisting infection/inflammation may be related to the rupture of membranes following amniocentesis, with the procedure itself possibly contributing to the multifactorial etiology of PPROM. Thus, control of inflammation is essential in order to prevent the risks associated with amniocentesis and other invasive procedures. Besides inflammatory mediators, such as IL-6 or prostanoids, like PGE_2_, involved in triggering uterine contractions, some effectors of inflammation, namely, MMPs, might be associated with poor outcomes of childbirth [7, 25]. Indeed, an increased AF expression of these extracellular matrix degrading enzymes has been connected with both PROM and PPROM [17, 26, 27], suggesting a role in the weakening and premature ripening of fetal membranes [13].

As some antibiotics, like Ampicillin and Cephalosporin, have been reported to directly decrease amniotic IL-6 and PGE_2_, their use as a tool in the prevention of amniocentesis complications has been suggested [28–30]. Previous studies have also demonstrated that oral administration of lactoferrin (LF), an 80 kDa iron-binding glycoprotein, is able to decrease IL-6 serum concentration [31]. In addition, a recent study has shown that vaginal administration of the compound four hours prior to amniocentesis reduces the AF concentration of the cytokine [20].

On the basis of these premises, in the present study we evaluated, for the first time, the effects of treatment with vaginal LF four hours prior to midtrimester genetic amniocentesis on the production of PGE_2_, active MMP-9, active MMP-2, and their inhibitors TIMP-1 and TIMP-2. Taken together, our results suggest that LF may play an anti-inflammatory role in the amniotic environment, thereby completing the scenario previously hypothesized on the basis of IL-6 findings [20]. We did, in fact, find decreased AF levels of PGE_2_, active MMP-9, and TIMP-1 in treated patients.

Considering that PGE_2_ directly triggers uterine contraction leading to abortion and preterm birth [25, 27] and that MMP-9 is involved in both weakening and ripening of fetal membranes [27], the use of LF as an anti-inflammatory medication may have potential clinical application in order to decrease the abortion rate following amniocentesis.

Although no experimental evidence exists, to the best of our knowledge, on amnion cells, we can hypothesize that LF might be able to decrease the production of PGE_2_ and active MMP-9 by acting at receptor and protein activation level, respectively. Indeed,* in vitro* experiments on macrophages have shown that the interaction of LF with a putative surface membrane receptor decreases the release of PGE_2_ [32]. At the same time, LF could also interfere with the activation cascade of MMP-9, since* in vitro* experiments have demonstrated its ability to reversibly chelate the zinc of the catalytic domain of MMP-2 [33]. However, further* in vitro* studies are required to confirm these hypotheses.

Nevertheless, the most intriguing outcome concerned the active MMP-2 that showed increased levels in response to LF treatment not accompanied by a concomitant change in TIMP-2, resulting in a mild increase in the MMP-2/TIMP-2 molar ratio, even if the ratio was <1, suggesting that the proteolytic activity of the enzyme was still modulated.

It is, therefore, tempting to speculate that LF can have a further protective role by increasing the levels of active MMP-2 following amniocentesis, which can drive the repair process of tissues. Indeed, in a previous study on an animal model of surgically induced membrane trauma, augmented AF production of MMP-2 was observed, modulating the repair process after the procedure [34]. In agreement with this hypothesis, we previously found that active MMP-2 was primarily expressed during the resolution phase of inflammation in inflammatory-based diseases by playing a role in the repair process [35, 36], most likely through the mitigation of the inflammatory signal and recruitment of immune cells [37]. However, few studies have suggested that MMP-2 can be involved in the setting of PROM and PPROM [11, 15], although a clear association with this pathological event has not been confirmed. Indeed, several studies reported no difference or even a decrease in active MMP-2 in the fetal membranes or AF in patients with PROM and PPROM and those with intact membranes [13, 14, 16, 17]. Although no complications were observed in our patients, further clinical studies on a more representative sample are mandatory to verify the nature of the protective role of LF administration in pregnancy.

Although the mechanism has not yet been identified, we can hypothesize that LF might potentiate the activation pathway of MMP-2 by increasing the activation rate mediated by its physiological activator, MT1-MMP [38]. However, without further* in vitro* experiments this hypothesis remains mere speculation.

Collectively taken, we can consider LF to have a two-fold beneficial effect: decreasing the inflammation by negatively influencing the production of cytokines and molecular effectors (e.g., active MMP-9) that play a role in membrane weakening and increasing the formation of active MMP-2, thus enhancing the repair process.

This study was not without its limitations. First, the relatively small sample size may have affected the reliability and clinical significance of our data. Second, the cross-sectional nature of the study, without assessing whether continuous treatment of patients with LF was able to steadily control the MMPs, TIMPs, and PGE_2_ production or decrease the preterm delivery, precluded the possibility of establishing any long term beneficial effects. To this purpose, a longitudinal approach would be more suitable, although we consider the present work as a good starting point to initiate longitudinal studies. Third, the presence of different AF levels of creatinine between controls and LF-treated patients without any evident difference in fetal maturity can be a further limitation of our study. Indeed, creatinine is usually associated with renal development and fetal maturity, with its levels increasing with gestational age and correlating with the weight of the fetus [39, 40]. However, to the best of our knowledge, there is no relation between inflammation and AF creatinine variations. Finally, we cannot exclude that the differences observed in creatinine levels might be due to patient selection or to unmeasured comorbidities that can affect maternal creatinine production or AF volume.

In conclusion, our results demonstrate that transvaginal LF is able to decrease the inflammatory response by modulating molecules involved in its course and, possibly, by potentiating the repair process in patients undergoing genetic amniocentesis. Since this glycoprotein is a natural component of the human organism, it represents a better choice compared to antibiotics in clinical conditions requiring administration of anti-inflammatory drugs. Therefore, transvaginal LF treatment may represent a safe, protective tool against the risk of abortion encompassed by amniocentesis.

## Supplementary Material

The supplementary material includes the extended version of the methods, along with the description of the basic principles, used for the determination of active MMP-9, active MMP-2 and PGE2.

## Figures and Tables

**Table 1:** Demographic and clinical characteristics of the controls, not treated with lactoferrin, and patients treated with lactoferrin 4 hours before amniocentesis (LF treated).

Characteristics	Controls (*n* = 57)	LF treated (*n* = 54)	*p* value
Maternal age (years)	36.5 (35.0–38.0)	37.0 (36.0–40.0)	0.061
Gestational age at amniocentesis (weeks)	16.99 (16.44–17.60)	16.91 (16.02–17.66)	0.576
Ethnicity (% (*n*))			0.063
White/Caucasian	93 (53)	81 (44)	
Other	7 (4)	19 (10)	
Pregestation BMI	21 (20–26)	21 (20–25)	0.687
Postgestation BMI	24 (20–27)	24 (20–26)	0.473
Primiparous (% (*n*))	65% (37)	55% (30)	0.367
Gestational age at delivery (weeks)	40.2 (39.3–40.7)	39.7 (38.5–40.7)	0.097
Creatinine (mg/dL)	0.83 (0.52–1.03)	1.17 (0.81–1.95)	*p* < 0.0001

Data are expressed as median (interquartile range). BMI: Body Mass Index; value of chi-square for ethnicity: *χ* ^2^(1) = 3.453; value of chi-square for primiparous: *χ* ^2^(1) = 0.812.
