# Supplementary material for: Vaginal Lactoferrin Modulates PGE2, MMP-9, MMP-2, and TIMP-1 Amniotic Fluid Concentrations
Source: Mediators Inflamm. 2016 Oct 31;2016:3648719. doi: 10.1155/2016/3648719 (PMC5107848; doi:10.1155/2016/3648719)
Supplement: Supplementary file 1 — The supplementary material includes the extended version of the methods, along with the description of the basic principles, used for the determination of active MMP-9, active MMP-2 and PGE2. [file 3648719.f1.pdf]

*Assay of active MMP-9*

The levels of active MMP-9 in the AF were determined by a commercially available activity assay kit (Fluorokine E, Human active MMP-9 fluorescent assat, R&D Systems, Minneapolis, USA). This is a fluorimetric assay designed to quantitatively measure enzyme activity, with a specific monoclonal antibody for human MMP-9 that has been pre-coated onto a black microtiter plate. Standard, a recombinant proMMP-9 in the range 16-0.25 ng/ml, and samples diluted 2 times with the calibrator diluent RD5-24 provided in the kit, were pipetted into the wells and incubated 2 hours at room temperature with gentle shaking. After four washing cycles, the activation reagent (APMA, p-aminophenyl mercuric acetate) was added to the wells of the standards in order to activate the proMMP-9, whereas APMA-free reagent diluent was added to all the well containing the samples, where the endogenous active MMP-9 had to be determined. The plate was then incubated 2 hours at 37°C in a humidified environment protected from light. Following four washing cycles, the fluorigenic substrate linked to a quencher molecule was added to all wells and the plate was incubated for 18 hours at 37°C in the dark. During this incubation, any active enzyme that was present in the samples cleaved the peptide bond between the fluorophore and the quencher molecule, generating a fluorescent signal that was read at excitation wavelength 340 nm and emission wavelength 405 nm in a Tecan Infinite M200 microplate reader (Tecan group Ltd, Männedorf, Switzerland). The amount of active MMP-9 in the sample was calculated by interpolation with the standard curve.

*Assay of active MMP-2*

Active MMP-2 concentration in the AF was determined by a commercially available activity assay kit (Matrix Metalloproteinase-2 Biotrak activity assay system, RPN2631, GE Heathcare, Milan, Italy). All reagents and standards were included in the kits. Briefly, 100 µl of standards, a recombinant human proMMP-2 in the range 4-0.125 ng/ml, and samples diluted 2 times with the assay buffer included in the kit were dispensed in wells of a microtiter plate pre-coated with anti-

human MMP-2 specific antibodies. After an overnight incubation at 4°C and four washing cycles, all the MMP-2 present in the sample was absorbed on the plate wells. Then, the standards were activated by adding 50 µl of 0.5 mM APMA whereas 50 µl of assay buffer were added in the samples, to quantify the endogenous active MMP-2. At the same time, 50 µl of detection reagent were pipetted in the wells. The detection reagent consisted in a mix containing a detection enzyme, a pro-form of urokinase whose natural activation sequence has been replaced with an artificial sequence recognized by MMP-2, and its specific chromogenic substrate. After 6 hours of incubation at 37°C, color development was read at 405 nm in a Tecan Infinite M200 microplate reader (Tecan group Ltd, Männedorf, Switzerland) and the amount of active MMP-2 present in the sample was calculated by interpolation with the standard curve.

***TIMP-1 and TIMP-2 assay***

Amniotic fluid concentrations of TIMP-1 and TIMP-2 were measured by using commercially available “sandwich” ELISA kits (Tissue inhibitor of metalloproteinases-1, RPN2611 and Tissue inhibitor of metalloproteinases-2, RPN2618; GE Healthcare, Milan, Italy) according to the manufacturer’s instructions. For the analysis of TIMP-1, amniotic fluids were diluted 40 times and the limit of detection was 1.25 ng/ml.  For the analysis of TIMP-2, amniotic fluids were diluted 2 times and the limit of detection was 3 ng/ml.

***$PGE_2$ assay***

Amniotic fluid $PGE_2$ was measured by a commercially available competitive enzyme immunoassay (Prostaglandin $E_2$ EIA kit-Monoclonal, Cat. No. 514010, Cayman Chemical Company, Ann Arrnor, USA) by following the manufacturer instructions.

Briefly, 50 µl of AF diluted 2 times with EIA buffer and $PGE_2$ standards in the range 1000-7.8 pg/ml were dispensed in duplicate in wells of a flat bottomed microtiter plate pre-coated with polyclonal goat anti-mouse IgG, along with 50 µl of tracer ($PGE_2$ linked to acetylcholinesterase) and 50 µl of $PGE_2$ mouse monoclonal antibody. For the determination of the maximum binding used for the calculations, 50 µl of EIA buffer followed by 50 µl of tracer and monoclonal antibody were dispensed in two wells in duplicate. One hundred microliters of EIA buffer was used as blank. After 18 hours of incubation at 4°C the plate was washed five times with wash buffer and 200 µl of Ellman's Reagent were added to each wells and incubated for 90 minutes in the dark with gentle shaking in order to develop the color. The plate was then read at 405 nm in a Tecan Infinite M200 microplate reader (Tecan group Ltd, Männedorf, Switzerland) and the amount of $PGE_2$ in the samples was determined by interpolation with the standard curve using a four-parameter logistic fit (GraphPad Prism version 6.00 for Windows, GraphPad Software, La Jolla California USA).
